# Polymorphisms in the *WNK1* Gene Are Associated with Blood Pressure Variation and Urinary Potassium Excretion

**DOI:** 10.1371/journal.pone.0005003

**Published:** 2009-04-04

**Authors:** Stephen Newhouse, Martin Farrall, Chris Wallace, Mimoza Hoti, Beverley Burke, Philip Howard, Abiodun Onipinla, Kate Lee, Sue Shaw-Hawkins, Richard Dobson, Morris Brown, Nilesh J. Samani, Anna F. Dominiczak, John M. Connell, G. Mark Lathrop, Jaspal Kooner, John Chambers, Paul Elliott, Robert Clarke, Rory Collins, Maris Laan, Elin Org, Peeter Juhanson, Gudrun Veldre, Margus Viigimaa, Susana Eyheramendy, Francesco P. Cappuccio, Chen Ji, Roberto Iacone, Pasquale Strazzullo, Meena Kumari, Michael Marmot, Eric Brunner, Mark Caulfield, Patricia B. Munroe

**Affiliations:** 1 Clinical Pharmacology and Barts and the London Genome Centre, William Harvey Research Institute, Barts and the London School of Medicine, Queen Mary University of London, London, United Kingdom; 2 Department of Cardiovascular Medicine, University of Oxford, Wellcome Trust Centre for Human Genetics, Oxford, United Kingdom; 3 Clinical Pharmacology and the Cambridge Institute of Medical Research, University of Cambridge, Addenbrooke's Hospital, Cambridge, United Kingdom; 4 Department of Cardiovascular Sciences, University of Leicester, Glenfield Hospital, Leicester, United Kingdom; 5 BHF Glasgow Cardiovascular Research Centre, Division of Cardiovascular and Medical Sciences, University of Glasgow, Western Infirmary, Glasgow, United Kingdom; 6 Centre National de Genotypage, Evry, France; 7 National Heart and Lung Institute, Faculty of Medicine, Imperial College London, London, United Kingdom; 8 Clinical Trial Service Unit and Epidemiological Studies Unit, University of Oxford, Oxford, United Kingdom; 9 Institute of Molecular and Cell Biology, University of Tartu, Tartu, Estonia; 10 Department of Cardiology and Institute of Molecular and Cell Biology, University of Tartu, Tartu, Estonia; 11 Centre of Cardiology, North Estonia Medical Centre, Tallinn, Estonia; 12 Department of Statistics, Pontificia Universidad Catolica de Chile, Santiago, Chile; 13 Clinical Sciences Research Institute, Warwick Medical School, Coventry, United Kingdom; 14 Department of Clinical & Experimental Medicine, Federico II University of Naples Medical School, Naples, Italy; 15 Department of Epidemiology and Public Health, University College London, London, United Kingdom; Innsbruck Medical University, Austria

## Abstract

*WNK1* - a serine/threonine kinase involved in electrolyte homeostasis and blood pressure (BP) control - is an excellent candidate gene for essential hypertension (EH). We and others have previously reported association between *WNK1* and BP variation. Using tag SNPs (tSNPs) that capture 100% of common *WNK1* variation in HapMap, we aimed to replicate our findings with BP and to test for association with phenotypes relating to *WNK1* function in the British Genetics of Hypertension (BRIGHT) study case-control resource (1700 hypertensive cases and 1700 normotensive controls). We found multiple variants to be associated with systolic blood pressure, SBP (7/28 tSNPs min-p = 0.0005), diastolic blood pressure, DBP (7/28 tSNPs min-p = 0.002) and 24 hour urinary potassium excretion (10/28 tSNPs min-p = 0.0004). Associations with SBP and urine potassium remained significant after correction for multiple testing (p = 0.02 and p = 0.01 respectively). The major allele (A) of rs765250, located in intron 1, demonstrated the strongest evidence for association with SBP, effect size 3.14 mmHg (95%CI:1.23–4.9), DBP 1.9 mmHg (95%CI:0.7–3.2) and hypertension, odds ratio (OR: 1.3 [95%CI: 1.0–1.7]).We genotyped this variant in six independent populations (n = 14,451) and replicated the association between rs765250 and SBP in a meta-analysis (p = 7×10^−3^, combined with BRIGHT data-set p = 2×10^−4^, n = 17,851). The associations of *WNK1* with DBP and EH were not confirmed. Haplotype analysis revealed striking associations with hypertension and BP variation (global permutation p<10^−7^). We identified several common haplotypes to be associated with increased BP and multiple low frequency haplotypes significantly associated with lower BP (>10 mmHg reduction) and risk for hypertension (OR<0.60). Our data indicates that multiple rare and common *WNK1* variants contribute to BP variation and hypertension, and provide compelling evidence to initiate further genetic and functional studies to explore the role of *WNK1* in BP regulation and EH.

## Introduction

Essential hypertension (EH), or high blood pressure (BP) ≥140/90 mmHg, is a major public health problem that contributes to millions of deaths worldwide every year due to coronary heart disease, stroke, and other vascular diseases [1], [2], [3], [4]. The disorder results from the complex interaction between multiple genes and environmental factors [5], [6], [7]. A major goal for hypertension research has been to identify the genes and mechanisms underlying the disorder in order to improve the prediction of those at risk and develop better anti-hypertensive treatments.

Some advances in hypertension genetics have been made through studies of rare Mendelian forms of hypertension, which have identified strong candidate genes for BP regulation and EH [8]. Mutations in *WNK1* (With No K-lysine kinase 1 [9], [10], MIM 605232), cause Pseudohypoaldosteronism type 2 (PHA2, MIM 145260) – a rare autosomal dominant disorder primarily characterised by early onset hypertension and hyperkalemia [8], [11]. In PHA2 patients, gain-of-expression mutations in *WNK1* cause hypertension. Conversely, heterozygous knock-out mice that lack *WNK1* expression have low BP, consistent with a gene-dosage effect of *WNK1* on BP [11], [12].

WNK1, a serine-threonine kinase regulating numerous ion channels involved in sodium and potassium transport [13], [14], [15], [16], [17], is ubiquitously expressed, with particularly high levels of expression in the kidney and cardiovascular system. *WNK1* maps to chromosome 12p13.3, spans ∼156 Kb of genomic DNA and encodes 29 exons [10], [18]. There are two major isoforms of *WNK1*, a kinase-active long isoform (*L-WNK1*) and a kinase-deficient kidney specific short isoform (*Ks-WNK1*). These two isoforms are under the control of alternative promoters located 5′ of exon 1 for *L-WNK1*, and in intron 4 for *Ks-WNK1* [19]. It is thought that hypertension in PHA2 patients may partially be the result of increased sodium reabsorption via *L-WNK1*/*Ks-WNK1* mediated up-regulation of the thiazide sensitive sodium chloride cotransporter (*SLC12A3* or NCCT) and the renal amiloride-sensitive epithelial sodium channel (ENaC, encoded by three genes: *SCNN1A*,*B and G*), and hyperkalemia by increased inhibition of the renal outer medullary potassium channel (*KCNJ1*) [13], [14], [15], [16], [17]. These findings together with the discovery that *WNK1*-deficient mice have low BP have highlighted the functional importance of *WNK1* in ion transport and BP regulation [12].

We and others have previously reported association between common variants in *WNK1* and human BP variation in adults. Using a tag SNP approach, association was found with a variant near the promoter and severity of hypertension in families from the British Genetics of Hypertension (BRIGHT) Study [20]. Furthermore, common *WNK1* variants were found to be associated with ambulatory BP in families representative of the general population [21]. Turner el. al. (2005) have also reported association between common *WNK1* variants and response to thiazide diuretics [22]. These studies prompted interest in further genetic studies exploring the role of WNK1 in BP regulation.

Since publication of these studies, additional SNP data have become available from the Haplotype Mapping Project (HapMap)[23]. Taking advantage of this gain in genetic information, our aim was to extend and replicate our findings between *WNK1* and BP variation and to further localise causative SNPs/regions within the gene using the British Genetics of Hypertension (BRIGHT) study case-control study, which provides substantial power for detecting susceptibility loci with moderate risks for disease.

## Methods

### MRC BRIGHT study case-control study

As part of the MRC BRIGHT study (http://www.brightstudy.ac.uk) hypertensive cases and normotensive controls of white European ancestry have been recruited for association testing. Case ascertainment and phenotyping has been described previously [24]. Briefly, cases have BP readings ≥150/100 mmHg based on one reading or ≥145/95 mmHg based on the mean of three readings and there is extensive phenotyping information from all individuals, including anthropometric data, plasma and urinary electrolytes, and diagnosis BP recordings. Further details of recruitment and phenotyping can be found online at www.brightstudy.ac.uk. Healthy, age and sex matched normotensive controls (BP readings ≤140/90 mmHg) had similar phenotyping, with the exception that plasma and urinary electrolytes were not measured. All subjects participated as volunteers and were recruited via hypertension registers from the MRC General Practice Framework in the UK. Ethics Committee approval was obtained from the multi- and local research committees of the partner institutes, and all participants gave written informed consent.

### The Estonian HYPEST sample collection

The Estonian participants were recruited during 2004–2007 across the entire country in the framework of the HYPEST sample collection (n = 1,823) targeting hypertension risk factors in the Estonian population (permissions no 122/13, 22.12.2003; 137/20, 25.04.2005 by Ethics Committee on Human Research of University of Tartu, Estonia). Hypertensive patients were recruited at the North Estonia Medical Centre, Tartu Estonia. Healthy (exclusion criteria; cardiovascular disease, diabetes, and antihypertensive treatment), normotensive individuals were recruited across the whole country. The majority of the HYPEST participants (n = 1,482) possess a documented history of multiple systolic blood pressure (SBP) and diastolic blood pressure (DBP) readings. For this study we defined cases (n = 596) as individuals with either blood pressure readings ≥160/100 mmHg based on the median of several measurements or under antihypertensive therapy. Controls (n = 650) were defined as having median blood pressure readings below 140/90 mm Hg. The quantitative association analysis of SBP and DBP (n = 1,284) included both untreated (n = 881) and treated individuals (n = 403).

### London Life Sciences Prospective Cohort Study (LOLIPOP)

This is a prospective study of 18,829 subjects (UK-based Indians, n = 12823 and white Europeans, n = 6006) investigating cardiovascular risk factors in certain sub-populations. For this study we selected white European individuals; 485 cases and 458 controls, drawn from the top and bottom 10% of the BP distribution. All blood pressure readings are off-medication.

### The Whitehall I study

The re-survey of the Whitehall study has DNA and blood pressure measurements recorded in middle age (1967–1970) and in old age (1997) on 5360 men. For this study, we selected white European men; 466 hypertensives and 536 controls, drawn from the top and bottom 10% of the BP distribution. The design and methods of Whitehall I as well as the characteristics of the participants have been described in detail elsewhere [25].

### The Olivetti Heart Study

The Olivetti Heart Study population is derived from the male workforce of the Olivetti factories of Pozzuoli (Naples) and Marcianise (Caserta), Italy. The general characteristics of the study and its methodological procedures have been previously described [26], [27]. The local ethics committee approved the study protocol, and informed consent was obtained from all participants. A total of 1085 individuals aged 25–74 years were examined in 1994–95: of these, 907 (83.6%) were seen again in 2002–04, of these 868 individuals had DNA available for genotyping at both time-points[26]. We analysed individuals at the 2002–04 time-point, cases were defined as individuals with BP readings of ≥145/95 mmHg, normotensive controls had BP readings of ≤140/90 mmHg.

### Whitehall II study

After ethical clearance the Whitehall study enrolled 10,308 subjects (3413 women) aged 35–55 working in the London offices of 20 civil service departments between 1985–1988. In this longitudinal study blood pressure was recorded at phase 1 (1985–1988), phase 3 (1991–1993), phase 5 (1997–1999) and phase 7 (2003–2004). DNA was stored from phase 7 of the study. For association testing with blood pressure and hypertension we selected individuals from Phase 5 as diabetes ascertainment and blood pressure medication records were most complete from this phase. For case control analyses, hypertensives were selected using blood pressure recordings of ≥145/95 mm Hg, or individuals taking anti-hypertensive medication or a physician diagnosis of hypertension. Normotensive controls were selected using blood pressure recordings ≤140/90 mmHg and not taking any anti-hypertensive medications.

### English Longitudinal Study of Ageing (ELSA)

After ethical approval the participants were drawn from around 12,000 respondents to the Health Survey for England (HSE) over three separate years (1998, 1999 and 2001) to provide a representative sample of the English population aged 50 and over. Each individual had a mean of three blood pressure measures taken when the participant was seated and antihypertensive medications were recorded, DNA was extracted from 5672 participants in wave 2 (2004). For association testing with blood pressure and hypertension we selected individuals from wave 2. Cases and normotensive controls were defined using the same criteria as the Whitehall II study.

### Tag SNP selection

The HapMap database was used to identify common polymorphisms across the *WNK1* genomic region [23]. Tag SNP selection was performed using Tagger (http://www.broad.mit.edu/mpg/tagger) [28]. *WNK1* SNP data were obtained from HapMap (Data Rel 21/phaseII Jul06, NCBI 35, dbSNP 125) using the *WNK1* chromosomal coordinates, chr12:732 992–888 219±10 kb, and the data used as input for Tagger. The original 8 tSNPs[20] and one additional SNP (rs3088353) located in the *L-WNK1* promoter were forced were forced in as tSNPs prior to the search. Additional tSNPs were selected to capture 100% of variation in HapMap with minor allele frequencies >0.05 and minimum R^2^ of 0.8.

### Genotyping

All SNP genotyping was performed using the Taqman assay developed by Applied Biosystems, followed by allelic discrimination using the ABI PRISM 7900HT Sequence Detection System and software (SDSv2.0, Applied Biosystems). Specific*WNK1* SNP Taqman probes and primers were obtained from Applied Biosystems Assay-by-DesignTM Service for SNP genotyping. Genotyping of individuals from the Whitehall study was performed at the Centre National de Génotypage, France, using the Taqman assay. The HYPEST sample was genotyped using Taqman, individuals from Whitehall II and the ELSA study were also genotyped using the Taqman assay at Geneservice UK.

### Statistical analysis of the BRIGHT study

Hardy–Weinberg equilibrium (HWE) was assessed by a χ*2* test. SNPs were dropped from all analyses if they were significantly out of HWE (rs11064519 HWE-p = 0.0001 and rs4980973 HWE-p = 0.0002 in control samples). The |D′| and *r* ^2^ measures of linkage disequilibrium were calculated using the program Haploview v4 [29].

All tests for association were performed with the statistical package R (http://www.r-project.org/)[30]. Logistic regression was used to test for association with EH and linear models for quantitative analyses. All analyses were adjusted for age, sex, body mass index (BMI) and centre of recruitment to allow for population stratification. The quantitative phenotypes were non-normally distributed and were analysed using naturally logged transformed data. Wald tests from a linear regression can be affected by non-normality and lead to inaccurate estimates of the variance-covariance matrix. Therefore, we also used an empirical estimate of the standard errors from 10,000 bootstrap samples to calculate the Wald tests. This approach selects random samples of size n (n = population size) with replacement from the original data, and repeats the sampling procedure a large number of times to provide information on the bias and variability of the parameter estimates. For each test of association, we repeated the selection procedure on 10,000 random samples and report the bootstrap estimate (effect) and 95% confidence intervals.

For single tSNP analyses, we tested for association under additive, dominant and recessive models. To control for multiple testing, global p-values were estimated using 10,000 permutations of the trait label to calculate the empirical distribution of the minimum p-value observed across all models for all SNPs. Multiple testing adjustments were applied separately to each trait, therefore only controlling for multiple testing within each trait. We only present results for tSNPs and haplotypes showing evidence for association after permutation testing with p<0.05.

Haplotype associations were explored using the R package HaploStats [31]. HaploStats estimates haplotype frequencies via the expectation-maximization (EM) algorithm and computes global and haplotype-specific score statistics for tests of association between a trait and haplotypes weighted by their posterior probabilities. Permutation p-values were computed in HaploStats using 10,000 permutations for 24 hour urine potassium excretion. For EH, SBP and DBP 10,000,000 permutations were used. In all haplotype analyses, we considered only those haplotypes with frequencies greater than or equal to 5% as separate independent variables, low frequency haplotypes with frequencies <5% were combined into a single group. To further explore the specific effects for individual low frequency haplotypes, we repeated the analysis to include haplotypes with frequencies ≥0.001. Haplotype specific Odds ratios (OR) and effect sizes were estimated using logistic regression for association with EH and linear models with the quantitative phenotypes in R weighted by their posterior probability [30], [31]. The most frequent haplotype was used as the baseline haplotype, with which effects of the other haplotypes were contrasted.

For the quantitative analyses with diagnosis BP (off-medication and therefore not confounded by the effects of drug treatment), data were available on 1183 cases and 1700 controls. These blood pressure readings were obtained from GP records at the time of their diagnosis. Mean BP levels from three readings for the 1183 cases and 1700 controls were used. For the biochemical phenotypes, analyses were performed in cases only, and these were on medication measures. No biochemical data were available for the control population. We did not have acidified urine for measuring urinary calcium or measures of serum potassium in the BRIGHT cases.

### Statistical analyses in replication cohorts and meta analyses

For quantitative association analysis of SBP and DBP, we corrected BP values for the effects of BP lowering therapies (where appropriate) using the method described by Tobin and colleagues [32], [33] -. for individuals on antihypertensive medication, we adjusted systolic and diastolic blood pressure measures by adding 15 mm Hg to systolic and 10 mm Hg to diastolic readings.

We then tested for association with BP variation and EH in each cohort separately using 10,000 bootstrap samples. To avoid multiple testing issues we analysed the data under a dominant model with the prior hypothesis that carriers of rs765250 [A] (A/A+A/G verses G/G) would have higher BP levels and be at increased risk for EH compared to G/G homozygotes, as determined from our primary analysis in the BRIGHT study case-control resource. As we are testing a specific hypothesis we report one-tailed p-values for the replication cohorts. We declare replication if the association is in the same direction with one-tailed p<0.05. Results were combined in a meta-analysis under a fixed effect model in R. Tests of heterogeneity were also evaluated using the I^2^ statistic [34]. If there was any statistical evidence for heterogeneity (p<0.05) then the analysis was repeated using a random-effects model, which includes a measure of variance between studies.

## Results

### *WNK1* variation

**Figure 1:**
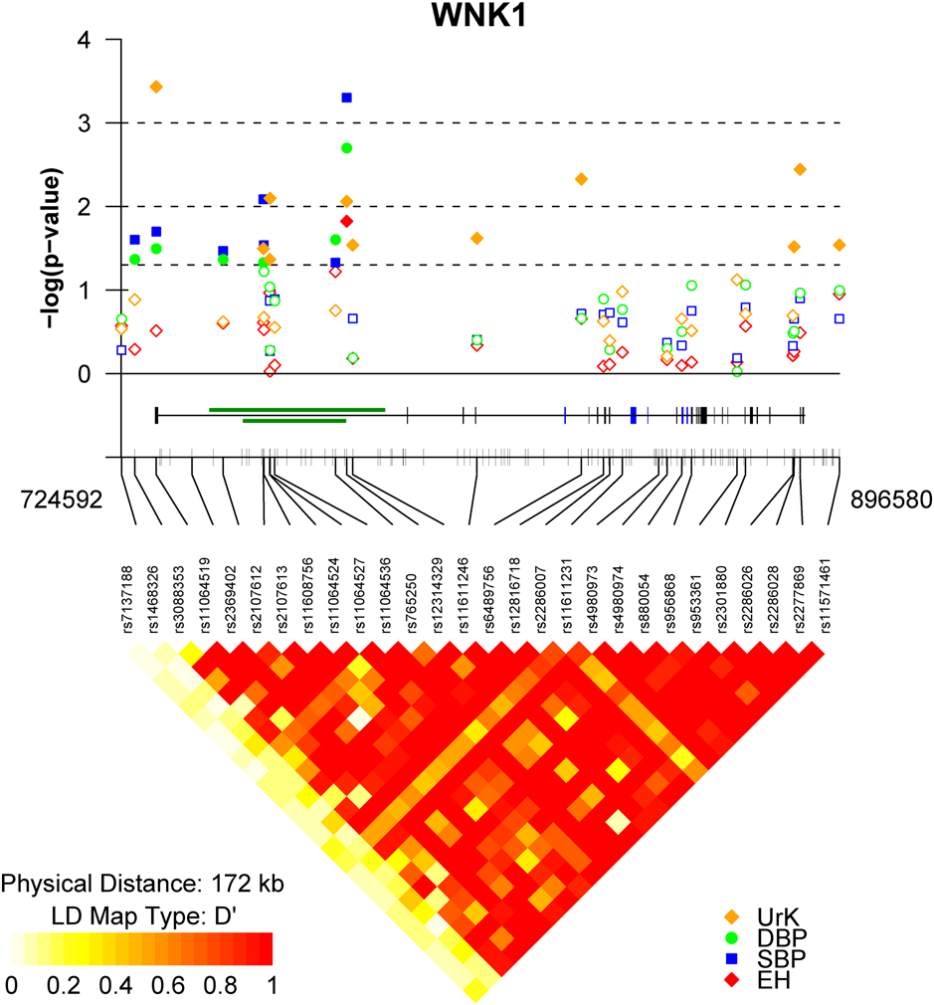
Association results and *WNK1* linkage disequilibrium. The diagram shows the summary results from the association analyses between *WNK1* tSNPs and essential hypertension (EH – red diamonds), blood pressure variation (SBP – blue squares, DBP – green circles) and urine potassium excretion (UrK – orange diamonds). The −log(10) of the best p-value are plotted on the y-axis against the physical position or each genotyped tSNP (x-axis), denoted by their dbSNP identification. Dotted lines represent P-value thresholds. Closed symbols represent significant associations (p<0.05). The tick marks along the x-axis also show the physical position of all common HapMap. The middle panel shows the genomic structure of the human *WNK1* gene and all known common variation across the *WNK1* genomic region. Exons are indicated by the vertical black bars and alternatively spliced exons by the blue boxes. The green boxes indicate the position of the PHA2 disease causing deletions. The lower panel represents the extent of linkage disequilibrium as measured by Lewontin's |D′| across the *WNK1* genomic region. |D′| varies between 0 (no disequilibrium) and 1 (maximum disequilibrium), represented by shades of white to yellow to red. White:|D′| = 0 and red:|D′| = 1. Strong LD (|D′|) exists between the most widely separated tSNPs, defining a single large haplotype block extending from tSNP 3 (rs3088353), located in the *WNK1* promoter, to tSNP 28 (rs11571461) located 3′ of *WNK1*. The plot was produced using a modified version of snp.plotter [49].

Twenty eight tagging SNPs (tSNPs) were identified that capture 100% of the common *WNK1* variation in HapMap (mean r^2^ = 0.9, range 0.8–1.0, Table 1, Figure 1). The average rate of success for each genotyped SNP was >95%. After genotyping, two SNPs (tSNP4 rs11064519 and tSNP19 rs4980973) were dropped from further analysis as they were significantly out of HWE (see Table 1). After exclusion of these tSNPs we were able to tag 97% of all common *WNK1* variation with r^2^>0.8 and 100% with r^2^>0.5.

**Table 1:** WNK 1 single nucleotide polymorphisms.

tSNP		gene position^a^	Chr12 nt position (bp)	Alleles	MAF^b^	HWE p Controls
1	rs7137188	5′	724592	C/T	0.48	0.1
2	rs1468326	5′	727762	C/A	0.11	0.8
3	rs3088353	5′	732901	A/C	0.46	0.9
4	rs11064519	i1	741472	C/G	0.34	0.0001
5	rs2369402	i1	748925	G/A	0.21	0.1
6	rs2107612	i1	758581	A/G	0.27	0.7
7	rs2107613	i1	758689	C/T	0.23	0.4
8	rs11608756	i1	760094	A/G	0.39	0.04
9	rs11064524	i1	760163	G/T	0.25	0.8
10	rs11064527	i1	761286	A/C	0.16	0.4
11	rs11064536	i1	775843	T/C	0.17	0.5
**12**	**rs765250**	**i1**	**778544**	**A/G**	**0.31**	**0.5**
13	rs12314329	i1	779992	A/G	0.08	0.4
14	rs11611246	i4	809741	G/T	0.22	0.3
15	rs6489756	i4	834767	A/G	0.47	0.7
16	rs12816718	i6	840061	G/T	0.15	0.6
17	rs2286007	ex8	841552	C/T	0.07	0.7
18	rs11611231	i8	844587	C/G	0.10	0.4
19	rs4980973	i9	853307	A/G	0.13	0.0002
20	rs4980974	i9	855289	A/G	0.44	0.6
21	rs880054	i10	858819	A/G	0.41	1
22	rs956868	ex13	861173	A/C	0.14	0.4
23	rs953361	i22	872068	C/T	0.40	0.7
24	rs2301880	i23	874098	C/T	0.24	0.7
25	rs2286026	i26	885434	A/G	0.41	0.9
26	rs2286028	i26	885730	C/G	0.20	0.7
27	rs2277869	I26	887171	C/T	0.16	1
28	rs11571461	3′	896580	A/G	0.07	0.3

a i: intron, e: exon, 5′: 5 prime; 3′: 3 prime.

b Minor allele frequency.

### Common *WNK1* variants are associated with blood pressure variation and essential hypertension in the BRIGHT study

We tested for association between 26 tSNPs with BP variation and EH in 1700 hypertensive cases and 1700 normotensive controls from the BRIGHT study. The demographic and biochemical characteristics of these individuals are shown in Table 2. For single tSNP analyses, we tested for association under additive, dominant and recessive models and corrected for multiple testing using permutation methods. Diagnosis BP measurements were used in the quantitative analyses, as these readings are off-medication and therefore not confounded by the effects of anti-hypertensive treatments.

**Table 2:** Characteristics of the BRIGHT Study Participants.

Variable	Subcategory	Controls	Cases	N	Normal range
Male/female		652/1048	652/1048	1700/1700	-
Age (years)		59.41 (±9.1)	59.5 (±9.1)	1700/1700	-
BMI (kg/m^2^) Median and IQR		25 (23–25)	27 (25–29)	1700/1700	-
**Blood Pressure**					
	clinic SBP(mmHg)	123.57 (±10.4)	155.62 (±21.2)*	1700/1700	-
	clinic DBP (mmHg)	76.8 (±6.9)	94.07 (±11.2)*	1700/1700	-
	diagnosis SBP (mmHg)	-	171.2 (±16.4)	1700/1183	-
	diagnosis DBP (mmHg)	-	103.75 (±8.1)	1700/1183	-
**Serum Biochemistry** ‡ * #					
	Sodium (mmol/L)	-	138.7 (±3.2)	1584†	135–144
	Chloride (mmol/L)	-	101.9 (±3.1)	1432†	97–108
	Calcium (mmol/L)	-	2.42 (±0.1)	1593†	2.2–2.6
	Ionised Calcium (mmol/L)	-	2.35 (±0.1)	1593†	2.16–2.5
**Urine biochemistry** * #					
	24 hour Sodium (mmol/24 hrs)	-	140.32 (±61.7)	1186†	100–250
	24 hour Potassium (mmol/24 hrs)	-	69.33 (±25.4)	1186†	35–100

Means±SD are presented unless otherwise stated.

* On medication.

# non-fasting.

‡ Serum potassium was not available.

† Cases only - Serum and urine biochemistry are not available for controls.

Multiple SNPs (7/26 tSNPs) located in the *WNK1* promoter and intron 1, were found to be significantly associated with BP variation under one or more of the models tested (Table 3 and Figure 1). Among the 7 BP associated SNPs, tSNP12, rs765250, located in intron 1, shows the strongest evidence for association; carriers of rs765250 allele A (A/A+A/G verses G/G) are associated with increased SBP (+3.1 mmHg, 95%CI:1.3,4.9, p = 0.0005) and DBP (+1.9 mmHg, 95%CI:0.7,3.2, p = 0.002). This same variant demonstrated evidence for association with EH, (OR:1.34, [95%CI:1.0–1.7], p = 0.015). The associations with SBP remained significant after permutation testing under the global null hypothesis “no SNP is associated with the trait of interest under any model”, but not with DBP or EH (SBP global permutation p = 0.02, DBP p = 0.06, EH p = 0.4).

**Table 3:** Single tSNP association analyses with EH, BP variation and 24 hour urinary potassium in BRIGHT.

Trait	tSNP	SNP ID	gene position^a^	Major^b^	Minor^b^	RAF^c^	Model^d^	Effect^e^	95% CI^e^	perm-p^e^	globa*L*-p^f^
**EH (Odds ratio)**	12	rs765250	i1	**A**	G	0.69	Dom	1.3	1.0,1.7	0.01	0.4
**SBP (mmHg)**	2	rs1468326	5′	C	**A**	0.11	Rec	5.1	0.6,9.2	0.02	
	3	rs3088353	5′	A	**C**	0.46	Rec	1.6	0.2,2.8	0.02	
	5	rs2369402	i1	G	**A**	0.21	Dom	1.2	0.1,2.2	0.03	
	6	rs2107612	i1	**A**	G	0.73	Add	1.2	0.2,2.0	0.008	
	7	rs2107613	i1	C	**T**	0.23	Dom	1.2	0.1,2.3	0.03	
	11	rs11064536	i1	**T**	C	0.83	Dom	3.1	0,6.1	0.05	
	12	rs765250	i1	**A**	G	0.69	Dom	3.1	1.3,4.9	0.0005	0.02
**DBP (mmHg)**	2	rs1468326	5′	C	**A**	0.11	Rec	3.1	0.01,5.8	0.04	
	3	rs3088353	5′	A	**C**	0.46	Rec	0.9	0.1,1.8	0.03	
	5	rs2369402	i1	G	**A**	0.21	Dom	0.8	0,1.5	0.04	
	6	rs2107612	i1	**A**	G	0.73	Add	0.6	0.01,1.2	0.05	
	7	rs2107613	i1	C	**T**	0.23	Dom	0.7	0.06,1.4	0.06	
	11	rs11064536	i1	**T**	C	0.83	Dom	2.5	0.2,4.8	0.02	
	12	rs765250	i1	**A**	G	0.69	Dom	1.9	0.7,3.2	0.002	0.06
**24 hour urine K+ (mmol/24 hour)**	3	rs3088353	5′	**A**	C	0.46	Dom	−5.9	−2.5,−9.2	0.0004	
	6	rs2107612	i1	**A**	G	0.73	Add	−2.6	−4.8,−0.3	0.03	
	8	rs11608756	i1	**G**	A	0.61	Add	−2.3	−4.3,−0.1	0.04	
	9	rs11064524	i1	T	**G**	0.25	Dom	−7.3	−12.1,−2.2	0.008	
	12	rs765250	i1	**A**	G	0.69	Add	−3.1	−5.4,−0.8	0.009	
	13	rs12314329	i1	A	**G**	0.08	Add	−4.3	−7.8,−0.5	0.03	
	14	rs11611246	i4	**G**	T	0.78	Add	−2.9	−5.3,−0.4	0.02	
	15	rs6489756	i4	A	**G**	0.47	Add	−3.2	−5.6,−0.9	0.005	
	27	rs2277869	i26	**T**	C	0.84	Add	−4.5	−7.6,−1.5	0.004	
	28	rs11571461	3′	A	**G**	0.07	Add	−4.7	−8.7,−0.5	0.03	0.01

a i: intron, 5′: 5 prime; 3′: 3 prime.

b Indicates major or minor alleles, bold indicates the allele increasing blood pressure.

c RAF: Risk Allele Frequency – refers to the allele increasing blood pressure or decreasing urinary potassium.

d Best Model; Add: Additive, Dom: Dominant, Rec: Recessive.

e Linear effect estimate, 95% confidence intervals for EH, diagnosis SBP, DBP and 24 hour urine potassium using 10 K bootstrap samples. P-vaules are based on 10,000 permutations.

f Global p-value based on 10,000 permutations - adjusting for testing multiple SNPs and multiple models.

### Replication of rs765250 association with blood pressure and essential hypertension

**Figure 2:**
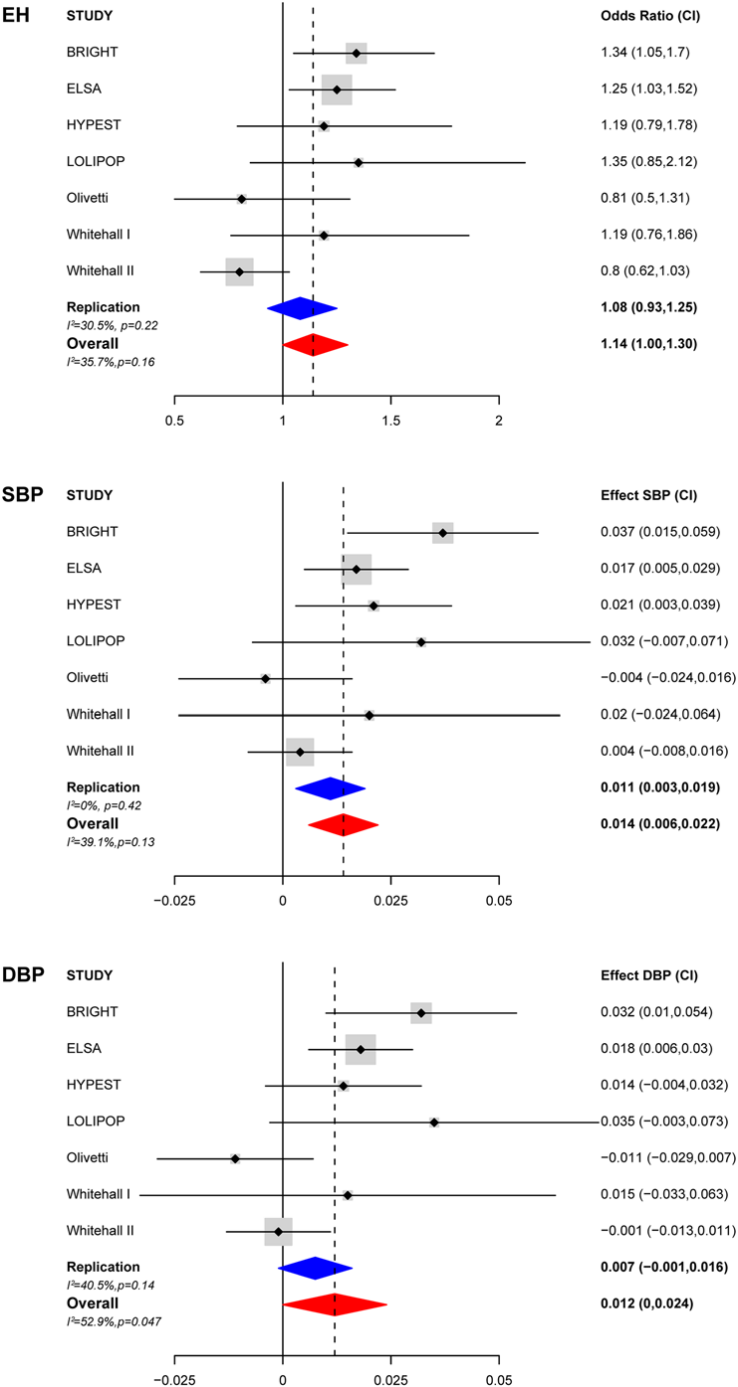
Meta-analysis plot showing the effect of rs765250 [A] carriers on risk for EH and blood pressure in 17,851 adults. A) Meta analysis of rs765250 with essential hypertension (EH), B) with systolic blood pressure (SBP), and C) with diastolic blood pressure (BBP). The size of the grey box is proportional to population size. Odds ratio/effect sizes and confidence intervals are from 10 K bootstrap samples. For the replication cohorts 90% confidence intervals are reported as all analyses were performed with the prior hypothesis that carriers of rs765250 allele A (A/A+A/G vs G/G) would have increased BP and be at increased risk for EH compared to G/G homozygotes. For EH and SBP, results were combined in a meta-analysis under a fixed effect model. Analysis of DBP revealed evidence for heterogeneity therefore results were combined in a meta-analysis using a random-effects model, which includes a measure of variance between studies.

The tSNP rs765250 demonstrated the strongest evidence of association with BP variation and EH. We genotyped this SNP in six additional populations totalling 14,451 individuals (HYPEST, Whitehall I, LOLIPOP, English longitudinal study of ageing (ELSA), Whitehall II and the Olivetti study) to confirm our findings,Table 4 shows selective demographic characteristics of each cohort. We tested for association with BP as a continuous trait and hypertension in all cohorts under a dominant model, with the prior hypothesis that carriers of rs765250 allele A (A/A+A/G verses G/G) would have higher BP levels and be at increased risk for EH compared to G/G homozygotes – as determined from our primary analysis in the BRIGHT study. The results for each trait are presented in Table 5 and Figure 2.

**Table 4:** Summary demographics of the replication case-control populations used in this study.

Cohort^a^	Case/Control	Male/Female	Age mean (SD)	BMI mean (SD)	SBP mean (SD)	DBP mean (SD)
BRIGHT	1700/1700	1304/2096	59.4(9.1)	26.3(3.6)	171.1(16.4)/123.6(10.4)	103.7(8.1)/76.8(6.9)
LOLIPOP	485/458	587/356	52.1 (11.3)	26.2(3.9)	154.8(17.9)/108.6(9.7)	95.4(7.8)/65.0(5.6)
Whitehall I	466/536	1002/-	48.2(4.8)	24.4(2.9)	163.8(14.7)/106.9(6.4)	102.2(9.3)/64.4(5.9)
HYPEST	596/650	397/849	48.8(13.6)	26.4(4.3)	144.2(18)/128(8.2)	87.6 (10.4)/80.6(6.3)
Whitehall II	4867	3620/1247	55.4 (5.9)	26.0 (3.9)	122 (15.9)	77.1(10.4)
ELSA	5422	2323/2745	63.6 (9.4)	27.8 (4.7)	136.1 (18.8)	75.7 (11.0)
Olivetti	971	971/-	51.5 (7.0)	27 (3.0)	129.7 (16.9)	84 (9.7)

a BRIGHT study summary demographics are included for comparison.

**Table 5:** Association results of rs765250 with essential hypertension and blood pressure variation per cohort and meta-analyses.

Study	Population	N^a^	Case/Control	EH		SBP		DBP	
				OR (CI)^b^	p-value^c^	Effect (se)^b^	p-value^c^	Effect (se)^b^	p-value^c^
**Original Study**	BRIGHT	3400	1700/1700	**1.3(1.0,1.7)**	**0.015**	**0.04(0.01)**	**0.0005**	**0.032(0.011)**	**0.002**
**Replication studies**	HYPEST	1246	596/650	1.2(0.8,1.8)	0.2	**0.02(0.01)**	**0.03**	0.01(0.01)	0.1
	LOLIPOP	943	485/458	1.3(0.8,2.1)	0.1	0.03(0.02)	0.09	0.03(0.02)	0.06
	Whitehall I	1002	466/536	1.2(0.8,1.9)	0.3	0.02(0.03)	0.2	0.01(0.03)	0.3
	ELSA	5422	2323/2745	**1.2(1.0,1.5)**	**0.03**	**0.02(0.01)**	**0.008**	**0.02(0.01)**	**0.005**
	Whitehall II	4867	1247/3620	0.8(0.6,1.0)	0.1	0.004(0.01)	0.3	−0.001(0.007)	0.4
	Olivetti	971	165/447	0.8(0.5,1.3)	0.5	−0.004(0.01)	0.4	−0.01(0.01)	0.2
**Meta-Analysis**	**Replication**	14451	5282/8456	1.1(0.9,1.2)	0.3	**0.1(0.004)**	**0.007**	0.007(0.004)	0.06
Test for heterogeneity				I^2^ = 30.5%,p = 0.22		I^2^ = 0%,p = 0.42		I^2^ = 40.5%,p = 0.14	
**Meta-Analysis** d	**Combined**	17851	6982/10156	**1.1(1.0,1.3)**	**0.04**	**0.01(0.004)**	**0.0002**	0.01(0.006)	0.06
Test for heterogeneity				I^2^ = 35.7%,p = 0.16		I^2^ = 39.1%,p = 0.13		I^2^ = 52.9%,p = 0.047	

a Effective number – maximum number of individuals available for analysis.

b Odds ratio and confidence intervals for EH using 10 K bootstrap samples. For the replication cohorts and meta analyses 90% confidence intervals are reported as all analyses were performed with the prior hypothesis that carriers of rs765250 allele A (A/A+A/G vs G/G) would be at increased risk for EH compared to G/G homozygotes.

c For the replication cohorts and meta analyses one-tailed p-values are reported.

d For EH and SBP results were combined under a fixed effects model. DBP showed evidence for heterogeneity and was analysed using a random-effects model.

Meta-analysis across all the replication cohorts (n = 14,451) revealed significant independent association with SBP (p = 0.007). There was a non-significant trend for association with DBP (p = 0.06). No association was seen with EH (OR:1.08, [95%CI:0.9,1.2], p = 0.33). To determine the overall significance and effect size in the samples studied, a meta-analysis was performed combining all cohorts including the original study population BRIGHT (n = 17,851). In the combined analysis, we confirmed association between rs765250 and SBP; carriers of rs765250 [A] were associated with an overall increase in SBP, with a mean effect on natural log transformed SBP of +0.01, [95%CI:0.006, 0.02], p = 2×10^−4^, equivalent to ∼2.0 mmHg. There was an association with increased risk for EH, OR:1.1 [95%CI:1.0,1.3], p = 0.04, and a trend for association with DBP p = 0.06.

### *WNK1* variants are associated with urinary potassium secretion in the BRIGHT study

As disturbances in electrolyte homeostasis accompany the development of high blood pressure in PHA2 patients carrying *WNK1* mutations, we hypothesised that variation in serum and/or urinary electrolytes would be indicative of altered *WNK1* function or expression, and this may help to identify polymorphisms that also affect BP. Therefore, we also explored associations with phenotypes related to *WNK1* function in the BRIGHT cases; measures of serum and urine biochemistry were not available for the BRIGHT controls. The biochemical characteristics of the hypertensive cases are presented in Table 2. We found multiple tSNPs spanning the length of the gene to be associated with variation in 24 hour urine K^+^ excretion (Table 3). These associations remained significant after correction for multiple testing (p = 0.01). The majority of cases were on medication at the time of phenotyping, therefore data were reanalysed including presence or absence of medication as a covariate in the regression analyses. After adjusting for medication, the evidence for association with 24 hour urine K^+^ remained (p≥0.001, data not shown). After permutation testing, no association was found with variation in serum Na^+^, Cl^−^, Ca^2+^ or urine Na^+^ excretion (data not shown).

### Haplotype analysis reveals striking associations with essential hypertension, blood pressure variation and urine potassium excretion

Using |D′| as a measure of linkage disequilibrium (LD) across the *WNK1* region, we identified a single large haplotype block extending from rs3088353 (tSNP3) located in the *WNK1* promoter, to rs11571461 (tSNP28) located 3′ of *WNK1* (Figure 1). *WNK1* haplotypes were constructed using 24/26 tSNPs that fall within this block (2 tSNPs were dropped as they were out of HWE – see *WNK1* variation). There are 8 common *WNK1* haplotypes with frequencies (f)>5% that account for ∼79% of all haplotypes in the BRIGHT case-control resource (Table 6); collectively the low frequency haplotypes account for the remaining ∼21% of haplotypic variation.

**Table 6:** *WNK1* Haplotypes.

		rs3088353	rs2369402	rs2107612	rs2107613	rs11608756	rs11064524	rs11064527	rs11064536	rs765250	rs12314329	rs11611246	rs6489756	rs12816718	rs2286007	rs11611231	rs4980974	rs880054	rs956868	rs953361	rs2301880	rs2286026	rs2286028	rs2277869	rs11571461
**Common**	**F**	**3**	**5**	**6**	**7**	**8**	**9**	**10**	**11**	**12**	**13**	**14**	**15**	**16**	**17**	**18**	**20**	**21**	**22**	**23**	**24**	**25**	**26**	**27**	**28**
1	0.15	1	1	1	1	1	1	1	1	1	1	1	**2**	1	1	1	**2**	1	1	**2**	1	1	1	1	1
2	0.13	**2**	1	1	1	**2**	1	**2**	1	1	1	1	1	**2**	1	1	1	1	**2**	1	1	1	1	1	1
3	0.12	**2**	1	1	1	1	**2**	1	1	1	1	**2**	**2**	1	1	1	**2**	1	1	**2**	1	1	**2**	1	1
4	0.12	1	**2**	**2**	**2**	**2**	1	1	**2**	**2**	1	1	1	1	1	1	1	**2**	1	1	**2**	**2**	1	1	1
5	0.10	1	1	1	1	1	1	1	1	1	1	1	**2**	1	1	1	1	**2**	1	1	1	**2**	1	**2**	1
6	0.06	1	**2**	**2**	**2**	**2**	1	1	1	**2**	1	1	1	1	**2**	1	1	**2**	1	1	**2**	**2**	1	1	1
7	0.06	**2**	1	**2**	1	1	1	1	1	**2**	**2**	1	1	1	1	1	**2**	1	1	1	1	1	1	1	**2**
8	0.05	**2**	1	1	1	1	**2**	1	1	1	1	**2**	**2**	1	1	**2**	**2**	1	1	**2**	1	1	**2**	1	1
**Low frequency**																									
14	0.008	**2**	1	1	1	1	**2**	1	1	1	1	1	**2**	1	1	1	**2**	1	1	**2**	1	1	**2**	1	1
15	0.008	1	1	1	1	1	1	1	1	1	1	1	**2**	1	1	1	1	**2**	1	1	1	**2**	1	1	1
18	0.004	**2**	1	1	1	1	**2**	1	1	1	1	1	**2**	1	1	**2**	**2**	1	1	**2**	1	1	**2**	1	1
19	0.004	1	1	1	1	1	1	1	1	1	1	**2**	**2**	1	1	1	**2**	1	1	**2**	1	1	1	1	1

All tSNPs are denoted numerically with reference to Table 2. Minor alleles “2” are highlighted in bold.

F: Frequency. Haplotype counts in cases and controls are estimates weighted by their posterior probabilities as determined by haplo.stats.

Table 7 shows the results of the haplotype analysis, it reveals striking associations with EH (global permutation p<10^−7^), BP variation (global permutation p<10^−7^), and 24 hour urine potassium excretion (global permutation p = 10^−4^). Across the BP related traits, the most significant associations were seen with the low frequency haplotype pool, which drive the highly significant global associations. The pool of low frequency haplotypes were found to be associated with decreased risk for EH, OR:0.6, [95%CI:0.5, 0.74], p = 4.82×10^−8^ and low BP (SBP-3.9 mmHg, [95%CI:−5.5,−2.5], p = 2.1×10^−7^; DBP −2.3 mmHg. [95%CI:−3.1,−1.4], p = 2.8×10^−7^). None of the common haplotypes were significantly associated with EH. However, three common haplotypes (haplotypes 3, 5 and 8) were associated with increased BP and five (haplotypes 2, 3, 4, 6 and 7) with variation in urine potassium excretion. The strongest association with BP variation was seen with haplotype 5, this accounts for ∼10% of all chromosomes in BRIGHT (SBP 2.4 mmHg, [95%CI:0.5, 4.2], p = 0.01; DBP 1.25 mmHg, [95%CI:0.2, 2.3] p = 0.02).

**Table 7:** Haplotype association analyses in BRIGHT.

Haplotype	Control^a^	Case^a^	F^a^	EH		SBP		DBP		24 hr Urine potassium^d^	
COMMON^a^				OR (95%CI)^b^	p-value^c^	Effect (95%CI)^b^	p-value^c^	Effect (95%CI)^b^	p-value^c^	Effect (95%CI)^b^	p-value^c^
1	477.5	513.9	0.15	reference	-	-	-	-	-	-	-
2	405.6	451.9	0.13	1.0(0.8,1.2)	0.8	1.7(−0.1,3.4)	0.06	0.9(−0.1,1.9)	0.09	**5.4(2.6,8.3)**	**9.8×10^−5^**
3	368.3	451.1	0.12	1.1(0.9,1.4)	0.2	**2.1(0.3,3.9)**	**0.02**	**1.2(0.2,2.2)**	**0.02**	**7.1(4.2,10.1)**	**5.7×10^−7^**
4	407.9	400.9	0.12	0.9(0.7,1.1)	0.4	−0.9(−2.7,,0.8)	0.3	−0.8(−1.8,0.2)	0.1	**6.1(3.2,9.1)**	**2.7×10^−5^**
5	307.3	406.0	0.10	1.1(0.9,1.4)	0.2	**2.4(0.5,4.2)**	**0.01**	**1.2(0.2,2.3)**	**0.02**	−0.0(−2.8,2.8)	0.98
6	193.1	205.3	0.06	1.0(0.8,1.3)	0.9	−1.3(−3.4,0.9)	0.2	0(−1.2,1.3)	0.9	**6.2(2.6,9.9)**	**4.7×10^−4^**
7	188.7	194.7	0.06	1.0(0.8,1.3)	0.9	−0.5(−2.7,1.8)	0.7	0.3(−1.0,1.6)	0.7	**9.1(5.3,13.1)**	**1.0×10^−6^**
8	163.3	195.2	0.05	1.0(0.8,1.4)	0.7	**2.5(0.1,4.9)**	**0.04**	**1.5(0.2,2.9)**	**0.03**	3.4(−0.2,7.3)	0.07
Low frequency pool	874.3	562.6	0.21	**0.6(0.5,0.7)**	**4.8×10^−8^**	**−4.0(−5.5,−2.5)**	**2.1×10^−7^**	**−2.3(−3.1,−1.4)**	**2.8×10^−7^**	**5.8(3.1,8.6)**	**1.89×10^−5^**

LOW FREQUENCY^a^	Control^a^	Case^a^	F^a^	OR (95%CI)^b^	p-value^c^	Effect (95%CI)^b^	p-value^c^	Effect (95%CI)^b^	p-value^c^	Effect (95%CI)	p-value
14	54.1	1.7	0.008	**0.03(0.006,0.1)**	**6.9×10^−6^**	**−15.9(−19.9,−11.7)**	**9.9×10^−13^**	**−9.2(−11.5,−6.7)**	**1.5×10^−12^**	-	-
15	53.8	1.6	0.008	**0.03(0.01,0.2)**	**3.9×10^−5^**	**−13.9(−18.1,−9.5)**	**1.5×10^−9^**	**−7.7(−10.2,−5.2)**	**5.4×10^−9^**	-	-
18	26.5	0.4	0.004	**0.01(0.0005,0.4)**	**0.01**	**−16.0(−21.5,−10.3)**	**1.5×10^−7^**	**−11.2(−14.4,−8.0)**	**1.0×10^−10^**	-	-
19	23.9	1.6	0.004	**0.06(0.01,0.3)**	**6.4×10^−4^**	**−17.9(−23.9,−11.6)**	**1.1×10^−7^**	**−10.2(−13.7,−6.5)**	**1.8×10^−7^**	-	-
**Global p-value** b					**<10^−7^**		**<10^−7^**		**<10^−7^**		**1×10^−4^**

a Haplotypes are numbered with reference to Table 4. Low frequency haplotypes are numbered with reference to Table 4 and Supplementary Table 1. Haplotype counts in cases and controls are estimates weighted by their posterior probabilities as determined by haplostats. F: Frequency.

b Linear effect estimate, 95% confidence intervals for EH, SBP, DBP and 24 hour urine potassium. The effect sizes are calculated by comparing each haplotype against haplotype 1: the most common haplotype or wild-type.

c Global p-value based on 10,000 permutations for 24 hour urine K+ and 10,000,000 for EH and SBP/DBP - adjusting for testing multiple haplotypes.

d Association with 24 hour urine potassium excretion was performed in cases only. Association analysis with urine potassium was not possible for the rare haplotypes. The associated rare haplotypes are almost uniquely distributed amongst the control population, for which as measures of 24 hour urine potassium excretion are not available.

To further explore the specific effects for individual low frequency haplotypes on BP variation and EH, we repeated the analysis to include haplotypes with frequencies ≥0.001 (∼minimum haplotype count of 5, Table 7, and Table S1). We identified 53 haplotypes with frequencies ≥0.001 (8 common haplotypes with f>0.05 and 45 low frequency haplotypes with 0.001≤f≤0.05). Analysis found 4 low frequency BP lowering haplotypes to drive most of the haplotype associations (haplotypes 14, 15, 18 and 19). Interestingly, these haplotypes were nearly unique to the control population, and as a result are associated with relatively large BP lowering effects when compared to the most common haplotype (>10 mmHg, Table 7, and Figure S1). For example, haplotype 14 was observed in at least 49 controls versus 1 case. These figures are counts of “phase-very-certain” haplotypes with posterior probabilities≥0.9, and differ from those in Table 7, which presents counts of all haplotypes weighted by their posterior probabilities, thus taking into account haplotype phase uncertainty. This low frequency haplotype (14), accounts for ∼1% of all haplotypes in BRIGHT, and was associated with low BP and decreased odds for hypertension (effect per copy of haplotype 14, OR: 0.03, [95%CI:0.006,0.1], p = 6.9×10^−6^, SBP −15.9 mmHg, [95%CI:−19.9,−11.7], p = 9.9×10^−13^, DBP −9.2 mmHg, [95%CI:−11.5,−6.7], p = 1.5×10^−12^); these association remained highly significant after permutation testing – global p<10^−7^ for EH and both BP traits.

## Discussion

We found multiple *WNK1* tSNPs and haplotypes to be significantly associated with BP, EH and urinary potassium excretion. The strong prior functional and genetic evidence for the role of *WNK1* in BP regulation [11] together with replication in additional populations, provides further support for the role of *WNK1* in BP regulation in both hypertensives and the general population. Although environmental effects, such as diet and drug treatment will confound the reported associations and may lead to inaccurate estimates of effect size, our data supports observations that *WNK1* regulates BP and K^+^ excretion *in vivo*, however, the association with K^+^ homoeostasis remains to be confirmed [16], [35].

In the BRIGHT study resource, the strongest tSNP association was seen between rs765250 and SBP. This association was replicated in additional populations, suggesting that the original association is unlikely to be a false positive. This association, however, was not replicated in all cohorts tested. Failure to replicate the effect of rs765250 on EH and BP in every population could be due to genetic heterogeneity across populations, small effect sizes or low power. Although the associations did not reach statistical significance in some of the individual cohorts, for the majority of populations, the direction of the effect was consistent with that seen in BRIGHT, with overlapping 95% confidence intervals. Encouragingly, in the combined meta-analyses, the evidence for association with SBP increased. Notably, this same SNP has previously been associated with ambulatory SBP and DBP in the families from the GRAPHIC study (min p = 0.001) [21], and more recently, with DBP gradient (p = 0.02) in children from the Avon Longitudinal Study of Parent and Children Study [36], lending further support to the reported findings.

In the single tSNP analyses, our primary associations with BP variation and EH were observed with variants located in the *WNK1* promoter regions and intron 1. In contrast to this, the tSNPS associated with urinary K^+^ excretion span the entire length of the gene. However, there is some overlap between those tSNPs associated with BP and variation in urinary K^+^. In particular, the variant rs765250, located in intron 1, which demonstrated the strongest evidence for association with EH and BP variation, is also associated with decreased in urinary K^+^ excretion. These novel genetic data correlate well with what is known about *WNK1* function, especially in relation to the primary phenotypes that characterise PHA2 - hyperkalemia and hypertension [37]. That is, we would expect true functional variants (or those in LD with these polymorphisms) to be associated with both BP and altered potassium excretion; this is what we observe.

Although there is some overlap between those tSNPs associated with BP and urinary potassium excretion (eg. 3/7 BP SNPs are also associated with variation in urinary potassium), not all BP associated variants were associated with urinary potassium and vice-versa. Furthermore, haplotypes associated with increased BP were not also associated with decreased urine potassium excretion. This discrepancy may represent complex interactions between *WNK1* polymorphisms and may also reflect the complexity of *WNK1* regulation and its role in electrolyte homeostasis.

There are two major isoforms of *WNK1*: *L-WNK1* and *Ks-WNK1*. *L-WNK1* is ubiquitously expressed, but *Ks-WNK1* has so far only been found to be expressed in the kidney [38]. Both *L-WNK1* and *Ks-WNK1* interact with each other to regulate common downstream targets involved in electrolyte homeostasis and BP regulation, via both kinase dependent and independent mechanisms [(e.g., sodium chloride co-transporter (*SLC12A3*), epithelial sodium channel (ENaC) and the renal outer medullary potassium channel (*KCNJ1*)]. These isoforms are under the control of alternative promoters – one located 5′ of the gene for *L-WNK1*, and the other in intron 4, controlling expression of *Ks-WNK1* [19]. Furthermore, both isoforms undergo tissue specific splicing and further variation is achieved by the use of two polyadenylation sites [18], [19], [39]. These data imply that there are multiple functional sites along the gene through which genetic variation could effect *WNK1* expression and function. Furthermore, it has been observed in some PHA2 patients carrying the *WNK1* deletion mutations, that the development of hyperkalemia may be separate from hypertension, and often precedes the development of high BP in these patients i.e., there may be no clear “cause and effect” relationship between the two phenotypes [40]. Therefore, it is possible that different *WNK1* polymorphisms, either singly or in combination, could contribute to the two different phenotypes. This could account for some of our observations and will need to be explored with further studies.

We found multiple tSNPs spanning the entire length of the gene and several haplotypes to be associated with the traits of interest, suggesting there may be multiple causal variants across the *WNK1* locus. Even though we used a comprehensive tSNP set that captured all known common *WNK1* variation in HapMap, HapMap does not contain a complete catalogue of all genetic variation, thus fine mapping will be required to identify the true causal variants. Interestingly, all common HapMap SNPs in strong LD (r^2^>0.8) with rs765250 and the other BP associated tSNPs, map to the *L-WNK1* promoter, the *Ks-WNK1* promoter located in intron 4 [19] and regions in intron 1 that span the sites of the PHA2 deletions (Figure S2), thus highlighting a few potential regions for targeted re-sequencing.

The most striking observations from our analyses were the identification of low frequency haplotypes with large BP lowering effects and their increased prevalence in the control population. Loss of *WNK1* function is deleterious, as demonstrated by homozygous knockout mice which are embryonic lethal [12]. On the other hand, heterozygous knockout mice have low blood pressure, and this is associated with decreased *WNK1* expression at the mRNA and protein level. Therefore, we can hypothesise that loss of function/expression mutations in *WNK1* would be selected against and be rare in the general population. More subtle mutations, however, that lead to decreased *WNK1* expression or function may be ‘protective’ against hypertension, and preserved at low frequencies in the general population. However, the effects of genetic drift should not be underestimated in terms of allowing slightly deleterious alleles to persist in human populations [41]. This may explain some of our observations. The ability of common tSNPs/haplotypes to capture rare functional variants has previously been demonstrated for the angiotensinogen (AGT) gene [42]. It is possible that our tSNP analysis set is capturing rare loss-of-function mutations that may be embedded in these low frequency haplotype backgrounds, thus highlighting the need to re-sequence individuals carrying these low frequency haplotypes.

Our data suggest that multiple common *WNK1* variants, with relatively weak effects, and multiple rare variants with large effects may be associated with blood pressure variation, and this should be explored further. Our findings are consistent with recent studies by Ji et al (2008) [43] and Tobin et al (2008) [44]. Both groups have performed a systematic analysis of the effect of variants in genes involved in renal salt handling on blood pressure and the development of hypertension. Many of which are regulated by *WNK1* – *SLC12A2*, *SLC12A1* and *KCNJ1*. Ji et al (2008) have identified rare variants in these genes that are associated with significantly lower blood pressure and protect from the development of hypertension in members of the Framingham Heart Study (FHS) [43]. In addition, Tobin et. al. (2008) have reported associations between common variants in these genes and blood pressure in families from the general population [44]. The findings suggest that both common and rare variants in genes responsible for some Mendelian disorders of hypertension and hypotension may also affect blood pressure variation in the general population. Our data lends further support to these observations.

The importance of rare variants to quantitative trait variability and susceptibility to disease has now been demonstrated for a number of other important phenotypes; including high density lipoprotein cholesterol (HDL-C), low density lipoprotein cholesterol (LDL-C) plasma triglycerides and body weight [45], [46], [47], [48]. We believe there is now compelling evidence to initiate further studies to identify functional *WNK1* variants that have a significant impact on BP variation, electrolyte homeostasis and risk of EH in the general population; thus taking an important step forward in our understanding of the pathogenesis of human essential hypertension.

## Supporting Information

Figure S1 Distribution of low frequency blood pressure lowering haplotypes The plot of systolic blood pressure (SBP)/diastolic blood pressure (DBP) values for each individual, showing the distribution of the associated low frequency haplotypes with posterior probabilities ≥0.9. The plot shows how the low frequency haplotypes are mainly found in low BP individuals.
(1.08 MB TIF)

Figure S2 The genomic structure of the human WNK1 gene is presented at the bottom of the panel. Exons are indicated by the vertical black bars and alternatively spliced exons by the red boxes. The yellow boxes indicate the position of the PHA2 disease causing deletions. For each of the blood pressure associated SNPs (red circles) located in intron 1, the r2 for each HapMap SNP with r2>0.8 is plotted on the y-axis against physical position (x-axis). The vertical dashed lines indicate the positions of the statistically similar SNPs.
(0.90 MB TIF)

Table S1 Haplotype association analysis
(0.04 MB XLS)

## References

[1] Kannel WB, Schwartz MJ, McNamara PM (1969). Blood pressure and risk of coronary heart disease: the Framingham study.. Dis Chest.

[2] MacMahon S, Peto R, Cutler J, Collins R, Sorlie P (1990). Blood pressure, stroke, and coronary heart disease. Part 1, Prolonged differences in blood pressure: prospective observational studies corrected for the regression dilution bias.. Lancet.

[3] Lewington S, Clarke R, Qizilbash N, Peto R, Collins R (2002). Age-specific relevance of usual blood pressure to vascular mortality: a meta-analysis of individual data for one million adults in 61 prospective studies.. Lancet.

[4] Petersen S PV, Rayner M, Leal J, Luengo-Fernandez R, Gray A (2005). European cardiovascular disease statistics 2005 edition.

[5] Ward R, Laragh JH, Brenner BM (1995). Familial aggregation and genetic epidemiology of blood pressure.. Hypertension : pathophysiology, diagnosis, and management. 2nd ed.

[6] Appel LJ, Moore TJ, Obarzanek E, Vollmer WM, Svetkey LP (1997). A clinical trial of the effects of dietary patterns on blood pressure. DASH Collaborative Research Group.. N Engl J Med.

[7] Stevens VJ, Obarzanek E, Cook NR, Lee IM, Appel LJ (2001). Long-term weight loss and changes in blood pressure: results of the Trials of Hypertension Prevention, phase II.. Ann Intern Med.

[8] Lifton RP, Gharavi AG, Geller DS (2001). Molecular mechanisms of human hypertension.. Cell.

[9] Xu B, English JM, Wilsbacher JL, Stippec S, Goldsmith EJ (2000). WNK1, a novel mammalian serine/threonine protein kinase lacking the catalytic lysine in subdomain II.. J Biol Chem.

[10] Verissimo F, Jordan P (2001). WNK kinases, a novel protein kinase subfamily in multi-cellular organisms.. Oncogene.

[11] Wilson FH, Disse-Nicodeme S, Choate KA, Ishikawa K, Nelson-Williams C (2001). Human hypertension caused by mutations in WNK kinases.. Science.

[12] Zambrowicz BP, Abuin A, Ramirez-Solis R, Richter LJ, Piggott J (2003). Wnk1 kinase deficiency lowers blood pressure in mice: a gene-trap screen to identify potential targets for therapeutic intervention.. Proc Natl Acad Sci U S A.

[13] Naray-Fejes-Toth A, Snyder PM, Fejes-Toth G (2004). The kidney-specific WNK1 isoform is induced by aldosterone and stimulates epithelial sodium channel-mediated Na+ transport.. Proc Natl Acad Sci U S A.

[14] Cope G, Murthy M, Golbang AP, Hamad A, Liu CH (2006). WNK1 affects surface expression of the ROMK potassium channel independent of WNK4.. J Am Soc Nephrol.

[15] Golbang AP, Cope G, Hamad A, Murthy M, Liu CH (2006). Regulation of the Expression of the Na/Cl cotransporter (NCCT) by WNK4 and WNK1: evidence that accelerated dynamin-dependent endocytosis is not involved.. Am J Physiol Renal Physiol.

[16] Lazrak A, Liu Z, Huang CL (2006). Antagonistic regulation of ROMK by long and kidney-specific WNK1 isoforms.. Proc Natl Acad Sci U S A.

[17] Subramanya AR, Yang CL, Zhu X, Ellison DH (2006). Dominant-negative regulation of WNK1 by its kidney-specific kinase-defective isoform.. Am J Physiol Renal Physiol.

[18] Shekarabi M, Girard N, Riviere JB, Dion P, Houle M (2008). Mutations in the nervous system–specific HSN2 exon of WNK1 cause hereditary sensory neuropathy type II.. J Clin Invest.

[19] Delaloy C, Lu J, Houot AM, Disse-Nicodeme S, Gasc JM (2003). Multiple promoters in the WNK1 gene: one controls expression of a kidney-specific kinase-defective isoform.. Mol Cell Biol.

[20] Newhouse SJ, Wallace C, Dobson R, Mein C, Pembroke J (2005). Haplotypes of the WNK1 gene associate with blood pressure variation in a severely hypertensive population from the British Genetics of Hypertension study.. Hum Mol Genet.

[21] Tobin MD, Raleigh SM, Newhouse S, Braund P, Bodycote C (2005). Association of WNK1 gene polymorphisms and haplotypes with ambulatory blood pressure in the general population.. Circulation.

[22] Turner ST, Schwartz GL, Chapman AB, Boerwinkle E (2005). WNK1 kinase polymorphism and blood pressure response to a thiazide diuretic.. Hypertension.

[23] Gibbs RA, Belmont JW, Hardenbol P, Willis TD, Yu F (2003). The International HapMap Project.. Nature.

[24] Caulfield M, Munroe P, Pembroke J, Samani N, Dominiczak A (2003). Genome-wide mapping of human loci for essential hypertension.. Lancet.

[25] Clarke R, Emberson JR, Parish S, Palmer A, Shipley M (2007). Cholesterol fractions and apolipoproteins as risk factors for heart disease mortality in older men.. Arch Intern Med.

[26] Strazzullo P, Barbato A, Galletti F, Barba G, Siani A (2006). Abnormalities of renal sodium handling in the metabolic syndrome. Results of the Olivetti Heart Study.. J Hypertens.

[27] Galletti F, Barbato A, Versiero M, Iacone R, Russo O (2007). Circulating leptin levels predict the development of metabolic syndrome in middle-aged men: an 8-year follow-up study.. J Hypertens.

[28] de Bakker PI, Yelensky R, Pe'er I, Gabriel SB, Daly MJ (2005). Efficiency and power in genetic association studies.. Nat Genet.

[29] Barrett JC, Fry B, Maller J, Daly MJ (2005). Haploview: analysis and visualization of LD and haplotype maps.. Bioinformatics.

[30] Ihaka R, Gentleman R (1996). R: A Language for Data Analysis and Graphics.. Journal of Computational and Graphical Statistics.

[31] Schaid DJ, Rowland CM, Tines DE, Jacobson RM, Poland GA (2002). Score tests for association between traits and haplotypes when linkage phase is ambiguous.. Am J Hum Genet.

[32] Tobin MD, Sheehan NA, Scurrah KJ, Burton PR (2005). Adjusting for treatment effects in studies of quantitative traits: antihypertensive therapy and systolic blood pressure.. Stat Med.

[33] Cui JS, Hopper JL, Harrap SB (2003). Antihypertensive treatments obscure familial contributions to blood pressure variation.. Hypertension.

[34] Higgins JP, Thompson SG, Deeks JJ, Altman DG (2003). Measuring inconsistency in meta-analyses.. Bmj.

[35] Fu Y, Subramanya A, Rozansky D, Cohen DM (2006). WNK kinases influence TRPV4 channel function and localization.. Am J Physiol Renal Physiol.

[36] Tobin MD, Timpson NJ, Wain LV, Ring S, Jones LR (2008). Common Variation in the WNK1 Gene and Blood Pressure in Childhood. The Avon Longitudinal Study of Parents and Children.. Hypertension.

[37] Gordon RD (1986). Syndrome of hypertension and hyperkalemia with normal glomerular filtration rate.. Hypertension.

[38] Delaloy C, Hadchouel J, Imbert-Teboul M, Clemessy M, Houot AM (2006). Cardiovascular expression of the mouse WNK1 gene during development and adulthood revealed by a BAC reporter assay.. Am J Pathol.

[39] O'Reilly M, Marshall E, Speirs HJ, Brown RW (2003). WNK1, a gene within a novel blood pressure control pathway, tissue-specifically generates radically different isoforms with and without a kinase domain.. J Am Soc Nephrol.

[40] Achard JM, Warnock DG, Disse-Nicodeme S, Fiquet-Kempf B, Corvol P (2003). Familial hyperkalemic hypertension: phenotypic analysis in a large family with the WNK1 deletion mutation.. Am J Med.

[41] Modiano G, Bombieri C, Ciminelli BM, Belpinati F, Giorgi S (2005). A large-scale study of the random variability of a coding sequence: a study on the CFTR gene.. Eur J Hum Genet.

[42] Zhu X, Fejerman L, Luke A, Adeyemo A, Cooper RS (2005). Haplotypes produced from rare variants in the promoter and coding regions of angiotensinogen contribute to variation in angiotensinogen levels.. Hum Mol Genet.

[43] Ji W, Foo JN, O'Roak BJ, Zhao H, Larson MG (2008). Rare independent mutations in renal salt handling genes contribute to blood pressure variation.. Nat Genet.

[44] Tobin MD, Tomaszewski M, Braund PS, Hajat C, Raleigh SM (2008). Common variants in genes underlying monogenic hypertension and hypotension and blood pressure in the general population.. Hypertension.

[45] Cohen JC, Kiss RS, Pertsemlidis A, Marcel YL, McPherson R (2004). Multiple rare alleles contribute to low plasma levels of HDL cholesterol.. Science.

[46] Cohen JC, Pertsemlidis A, Fahmi S, Esmail S, Vega GL (2006). Multiple rare variants in NPC1L1 associated with reduced sterol absorption and plasma low-density lipoprotein levels.. Proc Natl Acad Sci U S A.

[47] Ahituv N, Kavaslar N, Schackwitz W, Ustaszewska A, Martin J (2007). Medical sequencing at the extremes of human body mass.. Am J Hum Genet.

[48] Romeo S, Yin W, Kozlitina J, Pennacchio LA, Boerwinkle E (2009). Rare loss-of-function mutations in ANGPTL family members contribute to plasma triglyceride levels in humans.. J Clin Invest.

[49] Luna A, Nicodemus KK (2007). snp.plotter: an R-based SNP/haplotype association and linkage disequilibrium plotting package.. Bioinformatics.

