# Supplementary figures and images for: Polymorphisms in the WNK1 Gene Are Associated with Blood Pressure Variation and Urinary Potassium Excretion
Source: PLoS One. 2009 Apr 4;4(4):e5003. doi: 10.1371/journal.pone.0005003 (PMC2661139; doi:10.1371/journal.pone.0005003)

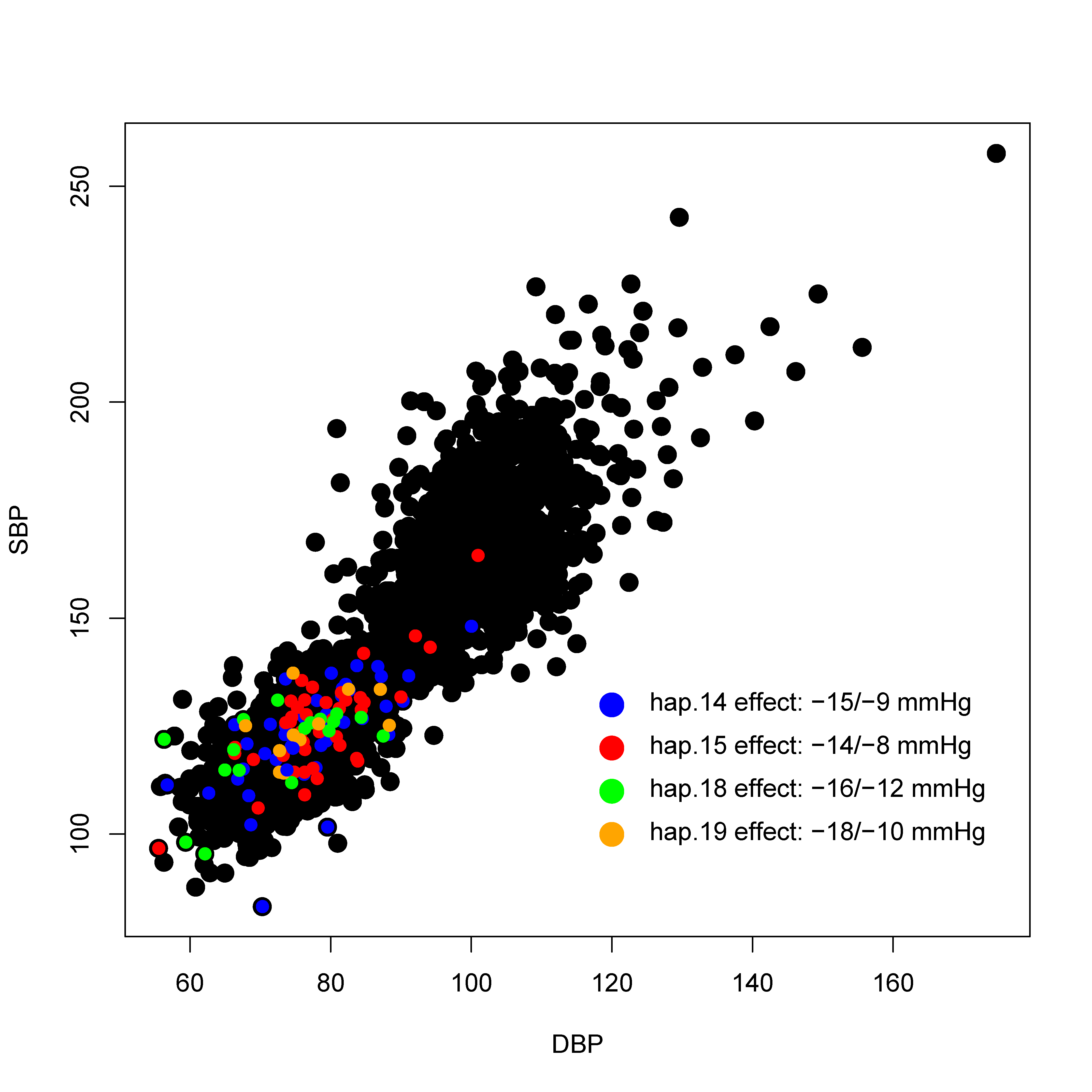

Supplement: Figure S1 — Distribution of low frequency blood pressure lowering haplotypes The plot of systolic blood pressure (SBP)/diastolic blood pressure (DBP) values for each individual, showing the distribution of the associated low frequency haplotypes with posterior probabilities ≥0.9. The plot shows how the low frequency haplotypes are mainly found in low BP individuals. (1.08 MB TIF) [file pone.0005003.s001.tif]

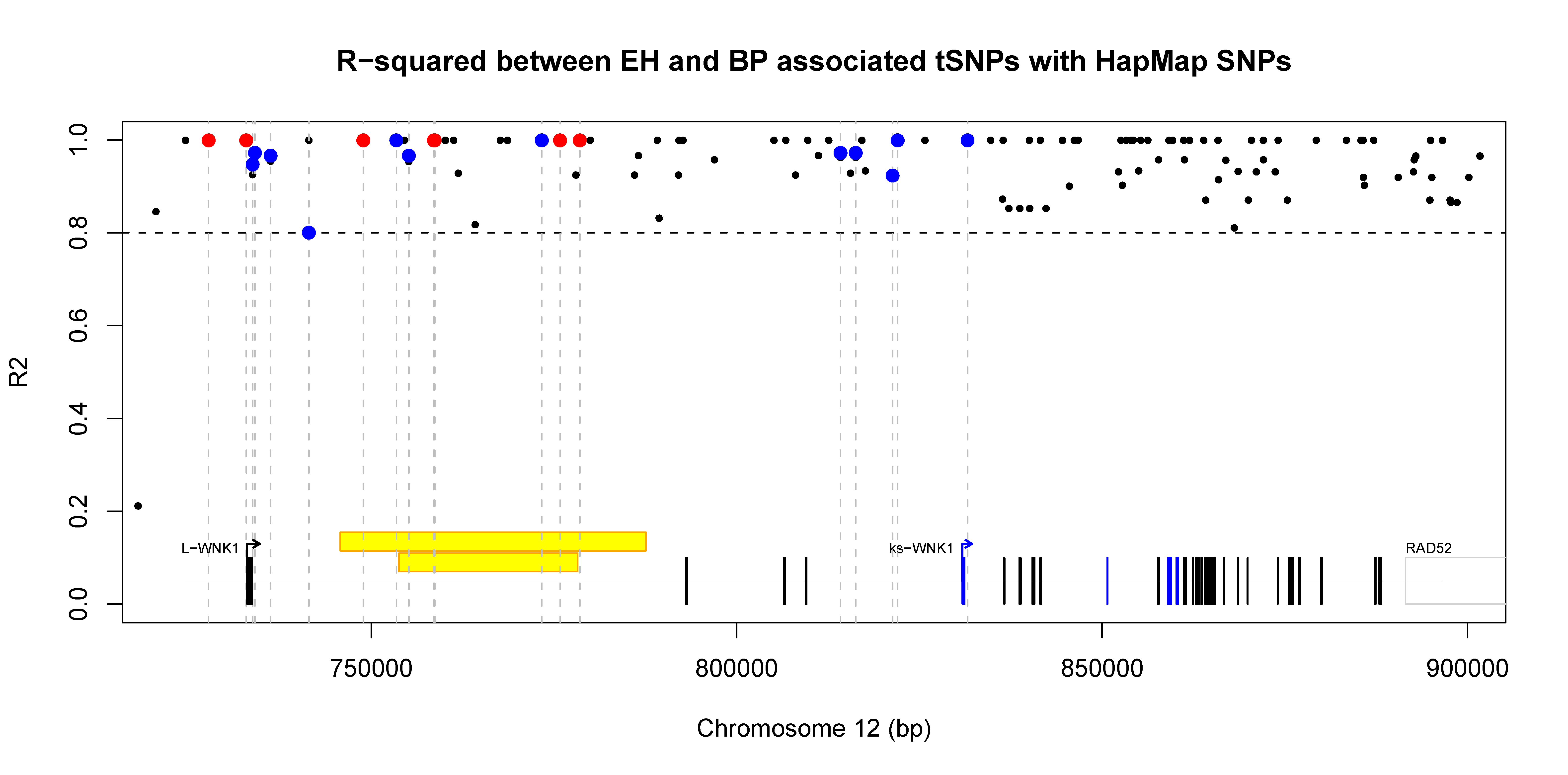

Supplement: Figure S2 — The genomic structure of the human WNK1 gene is presented at the bottom of the panel. Exons are indicated by the vertical black bars and alternatively spliced exons by the red boxes. The yellow boxes indicate the position of the PHA2 disease causing deletions. For each of the blood pressure associated SNPs (red circles) located in intron 1, the r2 for each HapMap SNP with r2>0.8 is plotted on the y-axis against physical position (x-axis). The vertical dashed lines indicate the positions of the statistically similar SNPs. (0.90 MB TIF) [file pone.0005003.s002.tif]
